# Increase in HDAC9 suppresses myoblast differentiation via epigenetic regulation of autophagy in hypoxia

**DOI:** 10.1038/s41419-019-1763-2

**Published:** 2019-07-18

**Authors:** Zhang Zhang, Liqiang Zhang, You Zhou, Liya Li, Jiangdong Zhao, Wen Qin, Zuolin Jin, Wenjia Liu

**Affiliations:** 1 General Surgery Department, Tang Du Hospital, Fourth Military Medical University, 710032 Xi’an, Shaanxi China; 2 State Key Laboratory of Military Stomatology & National Clinical Research Center for Oral Diseases & Shaanxi International Joint Research Center for Oral Diseases, Center for Tissue Engineering, School of Stomatology, Fourth Military Medical University, 710032 Xi’an, Shaanxi China; 3 State Key Laboratory of Military Stomatology & National Clinical Research Center for Oral Diseases & Shaanxi International Joint Research Center for Oral Diseases, Department of preventive dentistry, Fourth Military Medical University, 710032 Xi’an, Shaanxi China; 4 Department of Aerospace Biodynamics, Fourth Military Medical University, 710032 Xi’an, Shaanxi China; 5 State Key Laboratory of Military Stomatology & National Clinical Research Center for Oral Diseases & Shaanxi International Joint Research Center for Oral Diseases, Department of Orthodontics, School of Stomatology, Fourth Military Medical University, 710032 Xi’an, Shaanxi China

**Keywords:** Macroautophagy, Epigenetics

## Abstract

Extremely reduced oxygen (O_2_) levels are detrimental to myogenic differentiation and multinucleated myotube formation, and chronic exposure to high-altitude hypoxia has been reported to be an important factor in skeletal muscle atrophy. However, how chronic hypoxia causes muscle dysfunction remains unknown. In the present study, we found that severe hypoxia (1% O_2_) significantly inhibited the function of C2C12 cells (from a myoblast cell line). Importantly, the impairment was continuously manifested even during culture under normoxic conditions for several passages. Mechanistically, we revealed that histone deacetylases 9 (HDAC9), a member of the histone deacetylase family, was significantly increased in C2C12 cells under hypoxic conditions, thereby inhibiting intracellular autophagy levels by directly binding to the promoter regions of *Atg7*, *Beclin1*, and *LC3*. This phenomenon resulted in the sequential dephosphorylation of GSK3β and inactivation of the canonical Wnt pathway, impairing the function of the C2C12 cells. Taken together, our results suggest that hypoxia-induced myoblast dysfunction is due to aberrant epigenetic regulation of autophagy, and our experimental evidence reveals the possible molecular pathogenesis responsible for some muscle diseases caused by chronic hypoxia and suggests a potential therapeutic option.

## Introduction

Chronic high-altitude hypoxia contributes to the muscle atrophy observed in patients with pathologies associated with a hypoxic microenvironment, such as chronic obstructive pulmonary disease (COPD) and arteriosclerosis obliterans^1^. Some studies have reported that the hypoxia-induced inhibitory effect on muscle regeneration is a temporary and reversible process^2^, which seems to delay myogenic differentiation. By contrast, some studies have suggested that impaired regeneration under chronic hypoxia has long-lasting effects that may not be sufficiently reversed and results in muscle mass loss^3,4^; however, the underlying mechanism remains unclear.

Myogenesis is an essential step for muscle regeneration. In addition, myoblasts are required for this process because, by successfully differentiating and fusing with each other, they regenerate the characteristic multinucleated myofibers; they also play an important role in maintaining muscle structure and mass^5^. Some studies have shown that 1% O_2_ represses the myogenic differentiation of C2C12 (from a myoblast cell line)^2,6^, whereas a 3–6% O_2_ level can promote myogenesis^7^. Previous studies have reported that severe hypoxia negatively regulates myogenic differentiation by inhibiting MyoD or Myogenin in myoblasts in a manner that is dependent on^3^ or independent of^6,8^ hypoxia-inducible factor (HIF1α), a major contributor to the response to hypoxia signaling. Some studies have noted that myoblast differentiation is a reversible injury caused by hypoxia because myoblasts retain their capacity to proliferate or differentiate when normal oxygen levels are restored^2^. By contrast, other researchers believe that the effect of chronic hypoxia on muscle is perennial^3,4^. Furthermore, our laboratory previously confirmed that microenvironment-induced functional impairments can be transmitted to daughter cells in an epigenetically regulated manner^9^. However, the effects of chronic hypoxia on the functional alterations of myoblasts and the intrinsic underlying mechanism of its effects have been largely unexplored.

Acetylation of lysine residues on histones is a key process of the epigenetic regulation of DNA transcription^10^. The acetylation levels of lysine residues on histones are controlled by lysine acetyltransferases (KATs)/histone acetyltransferases (HATs) and histone deacetylases (HDACs). In the past several years, some studies have shown that HDACs respond to long-term hypoxia-induced gene transcriptional regulation^11^, adipocyte dysfunction^12^, and disease development^13,14^. Importantly, it has been demonstrated that histone deacetylase is involved in muscle-specific genes and in the regulation of muscle differentiation^15,16^. Therefore, we hypothesized that hypoxia might induce persistent changes in myoblasts via epigenetic regulation that causes subsequent muscle dysfunction.

In this study, we demonstrated that a high-altitude hypoxic microenvironment impaired the function of C2C12 in an epigenetically regulated manner. Mechanistically, we revealed that HDAC9 was significantly increased in the C2C12 due to hypoxia, thereby inhibiting intracellular autophagy by binding directly to the promoter regions of *Atg7*, *Beclin1*, and *LC3*. Decreased autophagy resulted in dephosphorylation of GSK3β and subsequent inactivation of the canonical Wnt pathway, impairing the myogenesis in the C2C12.

## Results

### Hypoxia inhibits the properties of C2C12 cells

**Fig. 1:**
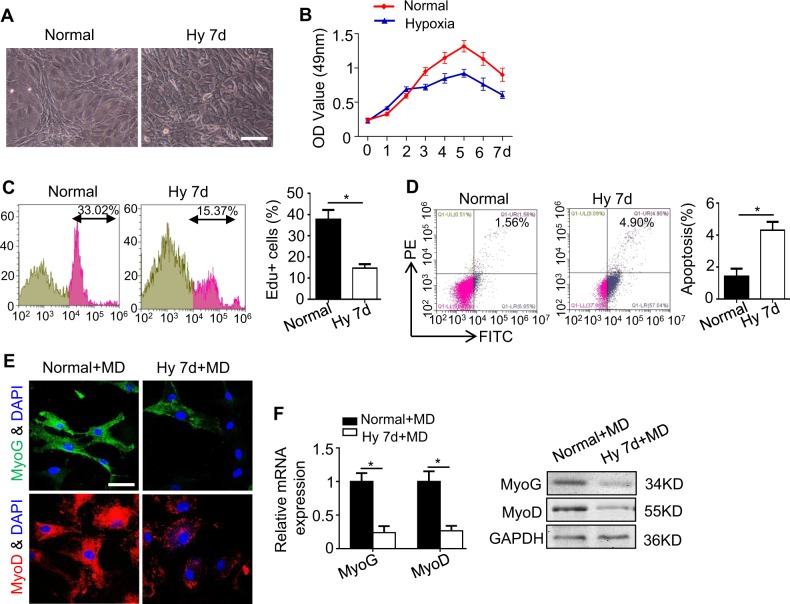
The properties of C2C12 cells decrease significantly under hypoxia. C2C12 cells were cultured under a normoxic or hypoxic microenvironment for 7 days. **a** The morphologies of the C2C12 cells grown under these two conditions were observed using an inverted microscope. Scale bar: 50 μm. **b**, **c** The viability and proliferation of the C2C12 cells under the two treatments were measured using MTT (**b**) and Edu (**c**) assays. **d** Apoptosis of the two C2C12 cells was examined by flow cytometry. **e** The C2C12 cells were cultured in myogenic differentiation medium (MD) under a normoxic or hypoxic microenvironment for 7 days, and MyoG (green)/MyoD (red)/nuclei (blue) were examined by immunofluorescence staining. Scale bar: 50 μm. **f** The myogenic genes of the C2C12 cells were detected by qRT-PCR and western blotting. The data are presented as the mean ± s.d. of triplicate samples from a representative experiment. *P *<* 0.05. **c**, **d** unpaired two-tailed Student’s *t*-test. **b**, **f** one-way analysis of variance (ANOVA)

To investigate the properties of the C2C12, myogenesis induction was performed. The C2C12 successfully formed myotubes and showed elevated expression of MyoG and MyoD (Supplementary Fig. 1), as indicated by qRT-PCR and western blotting. To investigate the effect of hypoxia on the function of myoblasts, the C2C12 cells were exposed to hypoxia (1% O_2_) or were maintained under standard conditions (21% O_2_). The morphology of the C2C12 was mostly unaltered under the hypoxic microenvironment; however, the number of granules was increased in the cytoplasm, and the nucleus was obviously visible (Fig. 1a). The viability and proliferation of the C2C12 were significantly reduced in the hypoxia group compared with the control groups, as shown by MTT and Edu analyses (Fig. 1b, c), indicating that although hypoxia promoted the viability of the C2C12 initially, the proliferation capacity was ultimately reduced after 7 days of exposure to hypoxia. Moreover, the number of apoptotic C2C12 was increased in the hypoxia group, as determined by flow cytometry (Fig. 1d).

We next examined the myogenesis of the C2C12 under normoxic and hypoxic conditions. The C2C12 cultured under hypoxic conditions for 7 days manifested severe inhibition of myogenesis, as indicated by reduced immunostaining and decreased expression of MyoG and MyoD (Fig. 1e, f), a finding consistent with previous reports^2,17^. Collectively, our results indicate that extremely low O_2_ levels inhibit the proliferation and differentiation capabilities of the C2C12 but promote apoptosis.

### Hypoxia inhibits the myogenesis in C2C12 cells via HDAC9

**Fig. 2:**
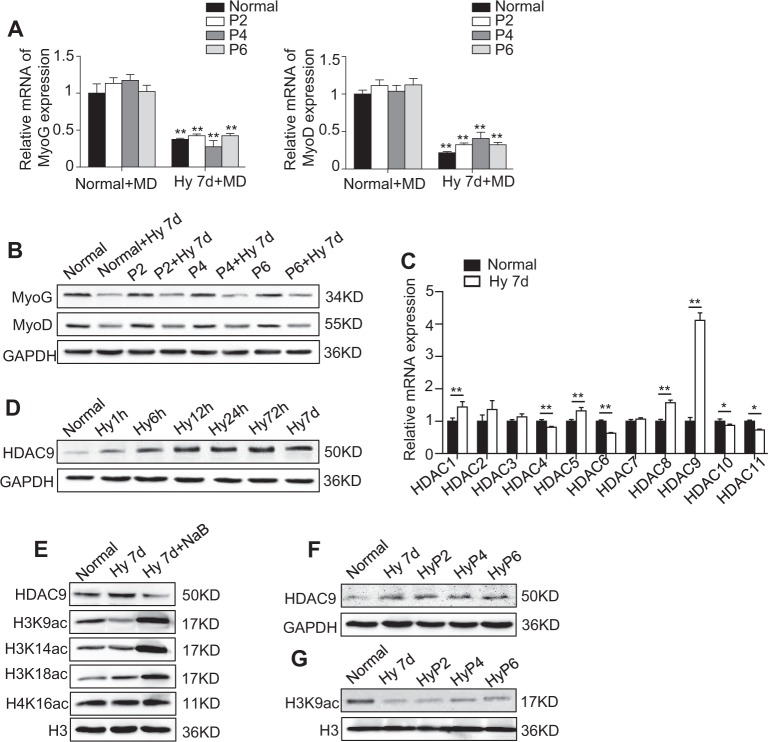
The effect of hypoxia on the disrupted function of C2C12 cells occurs in an epigenetic-dependent manner. **a**, **b** C2C12 cells were cultured in MD under normoxic or hypoxic conditions for 7 days as Normal (P1) or Hypoxia P1 (Hy), respectively, and then, both were continuously passaged six times under normoxic conditions. The expression of MyoG and MyoD in passages 2, 4, and 6 in the C2C12 cells was analyzed by qRT-PCR (**a**) and western blotting (**b**). **c** The C2C12 cells were cultured under normoxic or hypoxic conditions for 7 days, and the expression of the histone deacetylase family (*HDAC1-11*) was examined by qRT-PCR. **d** The expression levels of HDAC9 in the C2C12 cells at different time points after exposure to hypoxic stimuli were examined by western blotting. **e** The expression levels of HDAC9 and well-known histone targets of HDAC9 in normoxia or hypoxia with or without NaB treatment (the dose of NaB is 200 μM) in the C2C12 cells are shown. **f**, **g** The expression levels of HDAC9 and H3K9ac in different passages (normal, Hy7d, HyP2, HyP4, and HyP6) of the C2C12 cells were examined by western blotting. The data are presented as the mean ± s.d. of triplicate samples from a representative experiment. *P *<* 0.05, **P < 0.01. One-way analysis of variance (ANOVA)

Previous research has confirmed that the hypoxia-mediated inhibition of myogenic differentiation is reversible^2^. However, we observed lower expression levels of MyoG and MyoD in the C2C12 of passages 2, 4, and 6 under hypoxia compared with the same passages of C2C12 under normoxia (C2C12 cultured under hypoxic and normoxic conditions for 3 days were used as passage 1; Fig. 2a). The expression levels of MyoG and MyoD were almost the same with passaging, and they were lower than in the normal cells (Fig. 2b), indicating that the hypoxia-mediated inhibition of myogenesis in the C2C12 was consistent even when the cells were cultured under normoxic conditions for several passages.

Growing evidence suggests that the microenvironment changes the posttranscriptional modification of histones via acetylation^18–20^, which subsequently influences the function of cells^12^. Consequently, we compared the expression patterns of HDAC families between the C2C12 cultured under normoxic conditions and the C2C12 cultured under hypoxic conditions. The results showed that the expression levels of HDAC1, 2, 8, and 9 were continually elevated after exposure to hypoxia for 1 h, 24 h, and 7 days. In particular, the expression of HDAC9 increased nearly 5-fold in the hypoxia-exposed C2C12 compared with the normal control cells (Fig. 2c, Supplementary Fig. 2). Next, we focused on HDAC9 and examined the expression levels of HDAC9 at different time points under hypoxia. Western blotting analysis showed that the level of HDAC9 significantly increased under hypoxia, and this effect was also apparent in different passages (Fig. 2d, f). As hypoxia usually affects cell properties via hypoxia-inducible factors (HIFs)^3,21^, we also examined the expression levels of HIF1α and HIF2α at different time points in the cells under hypoxia. Western blotting analysis showed that HIF1α increased slightly after 1 h of hypoxia but then decreased continuously and almost disappeared after 72 h of hypoxia, and the expression pattern of HIF2α showed no correlation with the different time points of hypoxia (Supplementary Fig. 3). These results indicate that increased HDAC9 in the C2C12 has no close connection with HIFs; therefore, we did not study these factors in the other experiments.

HDAC9 has been found to prefer lysine 9, lysine 14, and lysine 18 of histone H3^22^, which it acetylates to regulate gene function; therefore, we examined the acetylation sites of histones 3 and 4 after blocking the expression of HDAC9. The results showed that, compared to trichostatin A (TSA), sodium butyrate (NaB) had a strong effect on the simultaneous inhibition of HDAC9 expression and promotion of H3K9 acetylation (Supplementary Fig. 4). Lysine residue H3K9 showed the opposite expression pattern to upregulated HDAC9. Furthermore, H3K9 was also hypoacetylated (more than 2-fold) in different passages of the hypoxia-C2C12, despite the enhanced level of HDAC9 (Fig. 2e–g), suggesting that hypoxia-induced HDAC9 elevation leads to histone deacetylation at lysine residue 9 of histone H3.

### Inhibition of HDAC9 rescues the hypoxia-induced impairment of myogenesis

**Fig. 3:**
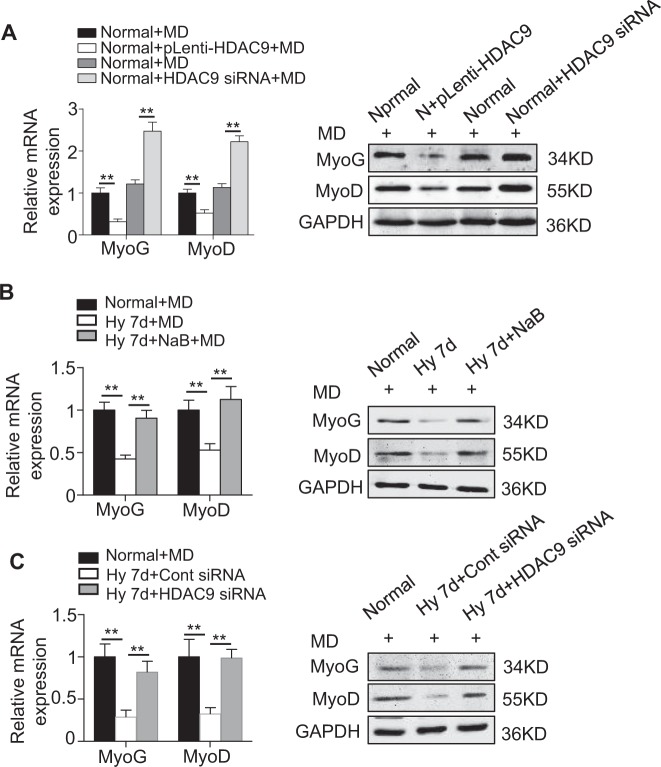
**Downregulation of the expression of HDAC9 by an inhibitor or siRNA rescues the inhibitory effect of hypoxia on C2C12 cell myogenesis.** **a** The regulation of HDAC9 expression through lentiviral vectors in the C2C12 cells is depicted. The expression of MyoG and MyoD was examined by qRT-PCR and western blotting at day 7 after myogenic induction. **b**, **c** To observe the effect of HDAC9 on the myogenesis in the C2C12 cells, C2C12 cells were treated with the HDAC inhibitor sodium butyrate (NaB) (**b**) or with HDAC9 siRNA (**c**). After myogenic induction for 7 days, the expression levels of MyoG and MyoD in the C2C12 cells were examined by qRT-PCR and western blotting. The data are presented as the mean ± s.d. of triplicate samples from a representative experiment. *P *<* 0.05, **P < 0.01. One-way analysis of variance (ANOVA)

The overexpression of HDAC9 in the C2C12 cultured under normoxic conditions significantly impaired their myogenesis. In contrast, the inhibition of HDAC9 expression in the C2C12 cultured under normoxic conditions partially enhanced their myogenesis, as confirmed by MyoG and MyoD expression (Supplementary Fig. 5, Fig. 3a). These data indicate that HDAC9 regulates the myogenesis in C2C12. We next investigated the therapeutic effect of downregulating HDAC9 on the functional recovery of the C2C12 cultured in hypoxia. We observed that the decreased expression levels of MyoG and MyoD induced by hypoxia were successfully rescued by NaB, suggesting that the therapeutic effect may be caused by the inhibited expression of the HDAC family that was induced by hypoxia (Fig. 3b).

Given that HDAC9 directly regulated the myogenesis in the C2C12 and that NaB is a broad-spectrum HDAC inhibitor, we next specifically downregulated the HDAC9 level in the C2C12 cultured in hypoxia. As expected, the decreased expression levels of MyoG and MyoD induced by hypoxia were also rescued after specifically downregulating HDAC9 (Fig. 3c). Collectively, these data indicate that the inhibited myogenesis of the C2C12 induced by hypoxia was likely due to the elevated HDAC9 level, and the downregulation of HDAC9 successfully rescued the C2C12 impaired by hypoxia.

### HDAC9 inhibits myogenesis via the epigenetic regulation of autophagy

**Fig. 4:**
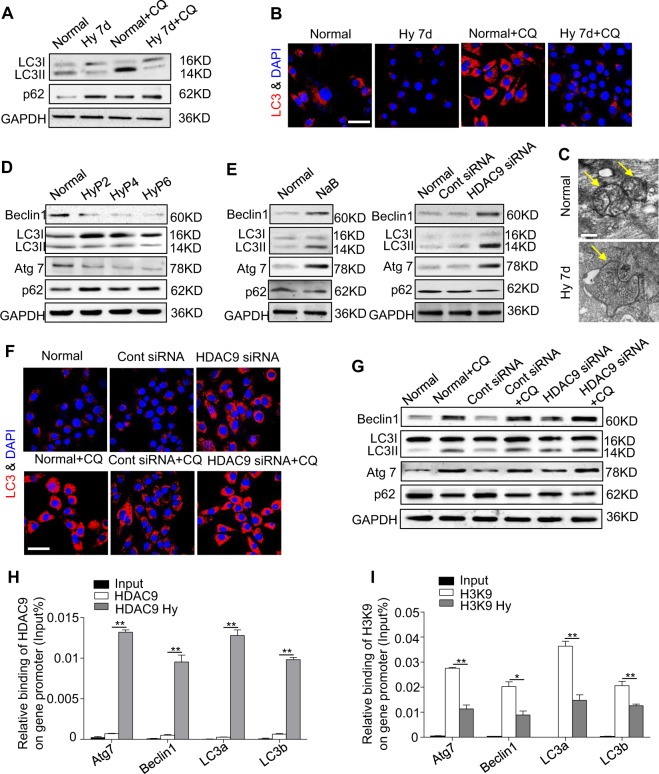
HDAC9 epigenetically regulates the autophagy level in C2C12 cells. **a** The expression levels of the autophagy-related proteins Beclin1, LC3I/II and specific cargo p62 after exposure to hypoxia for 7 days with or without chloroquine (CQ) were examined by western blotting. **b** The C2C12 cells were cultured under a normoxic or hypoxic microenvironment for 7 days with or without CQ. The LC3 (red)/nuclei (blue) in the C2C12 cells were analyzed by immunofluorescence staining. Scale bar: 50 μm. **c** To observe the autophagosomes, the C2C12 cells were cultured under normoxic or hypoxic conditions for 7 days and then observed using an electron microscope. Scale bar: 2 μm. **d** The expression levels of autophagy-related proteins in Hyp 2, Hyp 4, and Hyp 6 of the C2C12 cells were examined by western blotting. **e** The expression levels of autophagy-related proteins were examined by western blotting after treatment with NaB and HDAC9 siRNA. **f**, **g** Downregulation of HDAC9 in the normoxic C2C12 cells with or without CQ is shown. The LC3 (red)/nuclei (blue) in the C2C12 cells was analyzed by immunofluorescence staining, and the autophagy-related genes were analyzed by western blotting. Scale bar: 50 μm. **h**, **i** Chromatin was isolated from the C2C12 cells exposed or not to hypoxia and subjected to the chromatin immunoprecipitation assay using acetylated-histone H3K9 (Ac-H3K9) and HDAC9 antibodies. The IgG antibody was included as a control. The data are presented as the mean ± s.d. of triplicate samples from a representative experiment. *P *<* 0.05, **P < 0.01. One-way analysis of variance (ANOVA)

As autophagy is required to maintain cellular function and homeostasis under hypoxic conditions^23–25^, we next investigated the effect of hypoxia on autophagy in C2C12. The expression of LC3II was found in normal cells, and this expression was decreased after exposure to hypoxia for 7 days. In contrast, the expression of p62, which acts as specific cargo degraded by autophagy, was distinctly increased under the above hypoxic conditions compared with the control group (Fig. 4a). We next applied the autophagy-flux inhibitor chloroquine (CQ), which prevents lysosome degradation, thus increasing LC3II expression significantly when autophagy is active^26^. The CQ experiments showed that, in contrast to the cells cultured in normoxia, the cells cultured in hypoxia lacked the capacity for further autophagosome formation, as indicated by western blotting and immunostaining (Fig. 4a, b). Furthermore, we found fewer autophagosomes in the hypoxic cell group than in the normoxic cell group, as indicated by transmission electron microscopy (Fig. 4c). These results suggest that the autophagic activity was inhibited after long-term exposure of the cells to hypoxia. Moreover, the decline in the autophagic activity persisted from P2 to P6, showing the opposite effect of HDAC9. Accordingly, a lack of autophagy led to the accumulation of p62 in the passage 2 cells cultured under hypoxia but remained constant between P2 and P6 (Fig. 4d, Supplementary Fig. 6). Importantly, autophagy in the normal C2C12 was significantly activated and caused a decrease in p62 accumulation after the NaB treatment that downregulated HDAC9 in the presence or absence of CQ (Fig. 4e–g), as shown by western blotting and immunostaining analyses. These results indicate that HDAC9 and intracellular autophagy likely have a close relationship.

To examine whether HDAC9 directly regulates autophagy-related gene expression, we performed a chromatin immunoprecipitation (ChIP) assay. The results showed that HDAC9 was highly enriched at the promoters of *Atg7*, *Beclin1*, *LC3a*, and *LC3b* in the C2C12 (Fig. 4h), indicating that HDAC9 directly binds to the promoters of those autophagy-related genes. Accordingly, H3K9 was also highly enriched at the promoters of autophagy-related genes in the C2C12 (Fig. 4i), indicating that HDAC9 epigenetically regulates intracellular autophagy in C2C12.

**Fig. 5:**
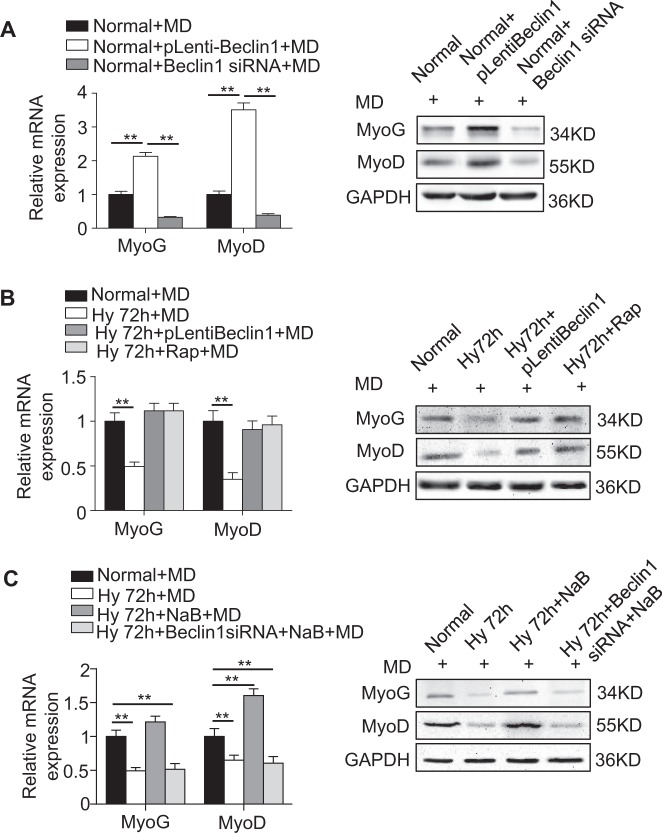
HDAC9 regulates myogenic differentiation of C2C12 cells likely through autophagy. **a** The regulation of Beclin1 expression in the C2C12 cells through the lentiviral vector and siRNA is depicted. The myogenesis-related genes MyoG and MyoD were examined by qRT-PCR and western blotting. **b** After activating autophagy in the hypoxic C2C12 cells by overexpression of Beclin1 or Rapamycin (Rap), the myogenesis-related genes MyoG and MyoD were examined by qRT-PCR and western blotting. **c** The C2C12 cells were cultured in MD and treated with NaB or Beclin1 siRNA under hypoxia for 72 h. The myogenesis-related genes MyoG and MyoD were examined in the C2C12 cells by qRT-PCR and western blotting. Normoxic C2C12 cells were used as a control. The data are presented as the mean ± s.d. of triplicate samples from a representative experiment. *P *<* 0.05, **P < 0.01. One-way analysis of variance (ANOVA)

Next, we tested whether the therapeutic effects of NaB or HDAC9 siRNA could rescue the hypoxia-impaired C2C12 directly by regulating autophagy. After Beclin1 was downregulated, the autophagy level decreased significantly and then suppressed the myogenic differentiation of C2C12, whereas overexpression of Beclin1 enhanced autophagy and myogenesis, as shown by qRT-PCR and western blotting (Supplementary Fig. 7, Fig. 5a). Then, we observed that activating autophagy in the C2C12 by upregulating Beclin1 or rapamycin could rescue the impaired myogenesis caused by hypoxia (Fig. 5b, Supplementary Fig. 8). More importantly, NaB could rescue myogenesis in the C2C12 after exposure to hypoxia, but this effect could be blocked by the downregulation of Beclin1 (Fig. 5c). Together, these results reveal that hypoxia reduced the myogenesis in the C2C12 mainly through HDAC9-mediated epigenetic inhibition of autophagy. We next assessed the mechanism by which autophagy regulates myogenesis.

### Autophagy regulates myogenesis in C2C12 cells by activating the Wnt/β-catenin pathway

**Fig. 6:**
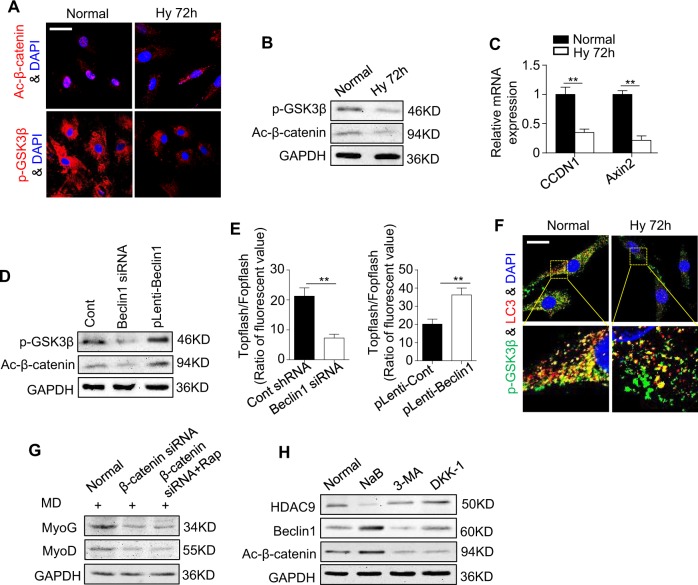
Autophagy regulates myogenic differentiation in C2C12 cells through regulation of the canonical Wnt pathway. **a**–**c** The expression levels of p-GSK3β and active-β-catenin were examined by immunofluorescence staining (**a**) and western blotting (**b**) after the cells were cultured in normoxia or hypoxia for 72 h. The downstream genes of the canonical Wnt pathway were analyzed by qRT-PCR (**c**). Scale bar: 50 μm. **d** The expression levels of p-GSK3β and active-β-catenin in the C2C12 cells were examined by western blotting after downregulation of Beclin1. **e** Activation of the canonical Wnt pathway was examined 48 h after transfection by luciferase assay. **f** Immunostaining showed overlapping of LC3 (red) and p-GSK3β (green) in the C2C12 cells cultured in normoxia and hypoxia for 72 h. Scale bar: 25 μm. **g** C2C12 cells were cultured in MD and transfected with control siRNA or β-catenin siRNA, and rapamycin was used to activate autophagy. Myogenesis-related genes were examined by western blotting after treatment for 72 h. **h** The C2C12 cells were treated with NaB, 3-MA, and DKK-1. The expression levels of HDAC9, Beclin1, and ac-β-catenin were examined by western blotting. The data are presented as the mean ± s.d. of triplicate samples from a representative experiment. *P *<* 0.05, **P < 0.01. **c** One-way analysis of variance (ANOVA). **e** unpaired two-tailed Student’s *t*-test

Wnts represent a class of secreted signaling proteins that modulate cell fate decisions, cell proliferation, and stem cell activity in a variety of embryonic and adult tissues. Many robust studies have reported that canonical Wnt signaling is important for regulating the muscle regeneration and myogenesis of myoblasts^27,28^. We also observed that the expression levels of phosphorylated GSK3β (p-GSK3β) and activated β-catenin (ac-β-catenin) were both decreased after exposure to hypoxia, as shown by immunostaining and western blotting. Furthermore, the levels of the *CCND1* and *Axin2* mRNAs, downstream intermediates in the Wnt/β-catenin pathway, were much lower in the hypoxic C2C12 (Fig. 6a–c), suggesting that the Wnt/β-catenin pathway was inactivated in hypoxia. More convincingly, we observed that activation of the canonical Wnt pathway by Wnt3a effectively rescued the impaired myogenesis in the C2C12 caused by hypoxia (Supplementary Fig. 9). These data indicate that inactivation of the Wnt/β-catenin pathway may contribute to the impaired function of the C2C12 caused by hypoxia.

Given that emerging studies have indicated that autophagy can contribute to regulation of the canonical Wnt pathway^29^, we first examined the expression of p-GSK3β and ac-β-catenin after regulating autophagy by lentivirus. Downregulation of *Beclin1* in the normoxic C2C12 impaired the Wnt pathway, mimicking the phenotype of hypoxic C2C12. In contrast, recovering *Beclin1* expression in the hypoxic cells reactivated the Wnt pathway, as confirmed by western blotting and TOPflash assays (Fig. 6d, e). These results suggest that the Wnt pathway was activated or inactivated depending on the level of autophagy. More importantly, we cultured C2C12 in normoxia and hypoxia for 72 h and visualized p-GSK3β and LC3 via confocal laser scanning microscopy. The results showed that LC3 could colocalize with p-GSK3β under both normoxic and hypoxic conditions. Notably, the merged images demonstrated that the colocalization of p-GSK3β and LC3 was lower in the hypoxic cell group due to the decreased expression levels of p-GSK3β and LC3 (Fig. 6f). Collectively, these results indicate that autophagy could directly regulate the canonical Wnt pathway, likely via phosphorylated GSK3β.

We then investigated whether autophagy regulates myogenic differentiation through the Wnt/β-catenin pathway. The results indicated that knockdown of the expression of β-catenin reduced the myogenesis in C2C12. Importantly, rapamycin could not recover the inhibited myogenesis after β-catenin was downregulated (Supplementary Fig. 10, Fig. 6g). Finally, to confirm the regulatory network of histone deacetylase, autophagy, and the canonical Wnt pathway, the C2C12 were treated with NaB, 3-MA, and DKK-1. Notably, NaB inhibited the expression of HDAC9 and promoted the expression of the remaining downstream genes, whereas the autophagy inhibitor 3-MA inhibited the expression of Beclin1 and ac-β-catenin. Furthermore, the canonical Wnt pathway inhibitor DKK-1 blocked only the expression of ac-β-catenin (Fig. 6h). Together, these results imply that the canonical Wnt pathway is under downstream regulation by HDAC9-mediated autophagy.

### HDAC9 epigenetically regulated autophagy is also observed in ischemic muscle atrophy

**Fig. 7:**
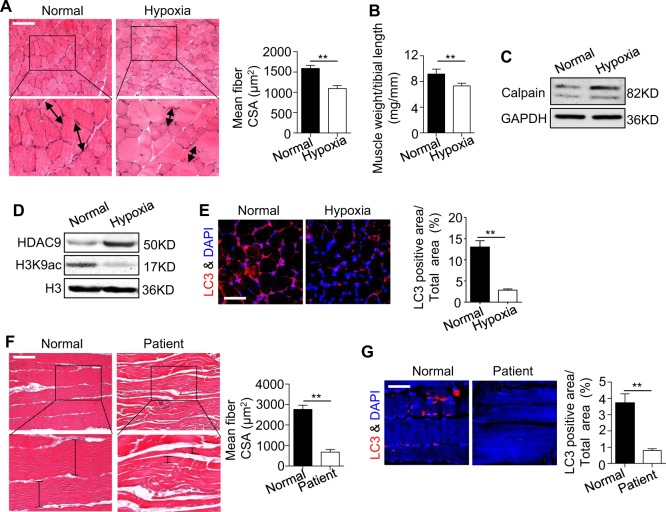
The autophagy that is epigenetically regulated by HDAC9 is also observed in the femoral artery (FA) ligation model and arteriosclerosis obliterans patients. We constructed a mouse single FA ligation model and harvested samples from both the control and surgery groups on day 21. **a** Hematoxylin and eosin staining (h&e) was used for visualizing the tissue from the mouse gastrocnemius muscle of the FA ligation model. The results from the quantitative analysis of the muscle fiber is indicated in the right panel. Scale bar: 200 μm. **b**, **c** The ratio of muscle weight/tibial length (**b**) and calpain expression (**c**) were analyzed in the control and ligation groups. **d** The expression levels of HDAC9 and H3K9 in the two groups were examined by western blotting. **e** The muscles samples were immunostained using anti-LC3 (red) and nuclear staining (blue, DAPI) to reveal the autophagy level. The results from the quantification analysis of the LC3^+^ cells is shown in the right panel. Scale bar: 100 μm. **f** H&E staining of the muscle in the normal control and arteriosclerosis obliterans patients is shown. The results from the quantification analysis are shown in the right panel. Scale bar: 200 μm. **g** The immunostaining analysis showed the expression of LC3 (red) and DAPI (blue) in the distal gastrocnemius muscle. Quantification of the LC3^+^ cells is indicated in the right panel. Scale bar: 100 μm. *n* = 8 for the mouse model and *n* = 2 for the patients. The data are presented as the mean ± s.d. of triplicate samples from a representative experiment. *P *<* 0.05, **P < 0.01. Unpaired two-tailed Student’s *t*-test

To further verify our investigation conducted in vitro, we first developed an ischemic muscle atrophy model in mice. The sections from mouse gastrocnemius muscles were examined histologically to evaluate the effects of chronic ischemic hypoxia on tissue integrity. The results showed normoxically grown muscle fibers had uniform size and shape with peripherally placed nuclei (Fig. 7a). Muscle tissue from mice exposed to ischemic hypoxia for 21 days showed severe atrophy of muscle fibers with irregularity in fiber size and more space between them. However, no necrotic fiber or cell splitting could be observed in any of the micrographs (Fig. 7a). Furthermore, with increased duration of exposure to ischemic hypoxia, the ratio of rat gastrocnemius muscle weight/tibial length decreased significantly, decreasing by 22% during 21 days of hypoxia exposure (Fig. 7b). Calpains are a member of the Ca^2+^-activated cysteine proteases that play important roles in cell motility, cell proliferation, and apoptosis^30^. Some investigations have suggested that calpains are activated during atrophy^31,32^. In our study, we observed that the expression level of calpain increased obviously after exposure to hypoxia for 21 days (Fig. 7c), suggesting that the muscle is atrophied after exposure to chronic ischemic hypoxia.

**Fig. 8:**
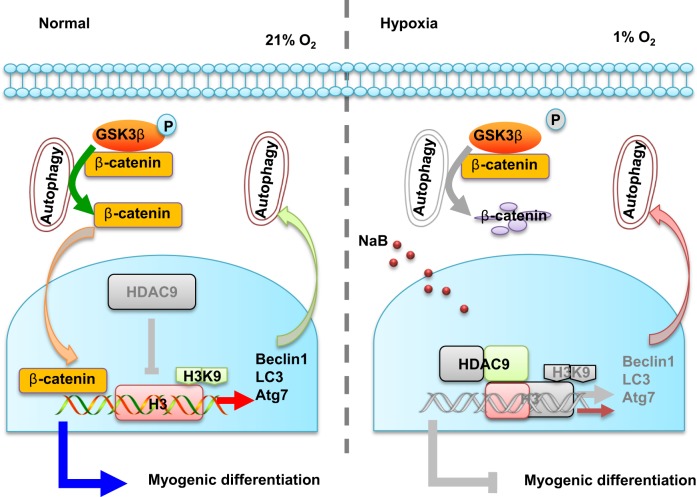
Schematic diagram depicts how hypoxia regulates myogenic differentiation of C2C12 cells and an epigenetics-guided therapeutic method. In the presence of normal oxygen, intracellular autophagy controls the phosphorylation of GSK3β and then promotes the transfer of β-catenin from the cytoplasm into the nucleus, which subsequently activates myogenesis-related gene expression. Under hypoxia, HDAC9 expression is increased due to the hypoxic microenvironment, and HDAC9 deacetylates H3K9 of autophagy-related genes and inhibits autophagosome formation. Insufficient autophagy subsequently results in dephosphorylation of GSK3β and inactivation of the canonical Wnt pathway, ultimately preventing myogenic differentiation. NaB treatment can partially rescue the impaired myogenic differentiation of the C2C12 cells caused by hypoxia

Given that HDAC9 expression was increased in the C2C12 after exposure to hypoxia and subsequently affected cell functions via the regulation of autophagy, we next tested whether HDAC9 regulates the level of autophagy in an ischemic hypoxia mouse model. The expression of HDAC9 increased but the level of H3K9ac decreased in the ischemic hypoxia group, as shown by western blotting (Fig. 7d). As expected, autophagy was dramatically lost in the hypoxia mouse model, as indicated by decreased LC3 levels (Fig. 7e), as in the C2C12 cultured in hypoxia. More importantly, we also observed the same muscle atrophy and decreased autophagy levels in patients with chronic muscle atrophy caused by arteriosclerosis obliterans (Fig. 7f, g). Collectively, our results indicate that hypoxia inhibits the regeneration of muscle likely via autophagy that is epigenetically regulated by HDAC9 (Fig. 8).

## Discussion

The effect of hypoxia on the myogenic process has been extensively studied. Several studies have provided credible data to show that less than 1% O_2_ level represses the proliferation and myogenesis of primary myoblasts and C2C12 in vitro^2,6,33^, raising the question of whether a long-term hypoxic microenvironment could permanently impair the myogenesis process. Some studies have demonstrated that myoblast differentiation is reversibly inhibited by hypoxia^2^, as myoblasts regain myogenesis capability when normal oxygen levels are restored. Conversely, our results revealed that the inhibitory effect on myogenesis caused by hypoxia was sustained in the C2C12 after six passages when normal oxygen was restored. We think the difference between the results from previous studies and those from our study may be due to the different culture conditions and observation methods. We previously reported that the microenvironment induces long-term effects on cell functions due to epigenetic regulation^5^. A previous report demonstrated that hypoxia-induced myogenic differentiation of embryonic stem cells is mediated by regulating HDAC6^34^. Another study has also shown that myogenesis under hypoxia is correlated with the deacetylation of histones associated with the myoD promoter^33,35–37^. Therefore, we first screened 11 HDACs. The results showed that HDAC9 was significantly upregulated in response to hypoxia. Downregulation of HDAC9 by applying HDAC9 siRNA or the HDACs inhibitor NaB recovered the inhibitory effect of hypoxia on C2C12 myogenesis. Thus, we showed that chronic exposure to severe hypoxia-induced muscle dysfunction or mass loss likely through epigenetic regulation similar to that observed in hypoxia-induced Alzheimer’s disease^38,39^, pulmonary hypertension^40^, and cardiac tissue fibrosis^41^. Notably, we observed that the autophagy of the C2C12 was first enhanced after exposure to hypoxia and subsequently decreased after 6 h in hypoxic culture. However, the expression of HDAC9 was continuously increased after exposure to hypoxia. Therefore, we infer that HDAC9 likely regulates autophagy during long-term hypoxic exposure.

Autophagy^24,42^ is a primary survival pathway for recycling cellular material during periods of nutrient starvation and in response to hypoxia, endoplasmic reticulum stress, and other stresses, and it is essential for maintaining tissue homeostasis. Importantly, dysregulated autophagy is observed in the pathogenesis of myopathies and muscular dystrophies^43,44^. A recent study demonstrated that autophagy was significantly enhanced in response to short-term intermittent hypoxia-induced atrophy of skeletal muscle^45^. In that study, LC3II protein was increased (2.4-fold) in limb muscle after 4 days of intermittent exposure to hypoxia. However, the effect of hypoxia-induced autophagy on myoblast differentiation has not been well elucidated. In the present work, we found that long-term exposure to hypoxia reduced the level of autophagy in the C2C12 and that autophagy was suppressed with cell passages. However, we noticed that the level of autophagy in the C2C12 was increased significantly under a short-term (<6 h) hypoxic stimulus and then gradually declined with prolonged hypoxia (data not shown). This finding is in agreement with previous reports showing that acute exposure to hypoxia increases the expression of markers of autophagy^45,46^. Interestingly, we noted that the inhibition of autophagy by Beclin1 siRNA blocked the effect of the NaB on rescuing the function of the C2C12 in hypoxia. Importantly, we confirmed that HDAC9 directly bound to the promoters of *Atg7*, *Beclin1*, *LC3a*, and *LC3b* in C2C12. The abovementioned results suggest that autophagy, which is epigenetically regulated by HDAC9, is required to maintain the function of C2C12 in a chronic hypoxic microenvironment.

Some studies have shown that Wnt/β-catenin signaling is critical for the regulation of muscle development and the myogenic differentiation of myoblasts^47–49^. Conditional depletion of β-catenin in mice led to reduced muscle mass and fewer myofibers, as shown by Pax7-positive muscle progenitor cell staining^50^. Previous reports have demonstrated that Wnt/β-catenin signaling is essential for the multiple steps of myogenesis^28,48,51,52^, but few reports have indicated that Wnt/β-catenin signaling is not required during muscle regeneration^53^. In the present study, we revealed that autophagy maintained the functions of myoblast cell lines by regulating the phosphorylation of GSK3β, which is an important regulator for modulating β-catenin nuclear translocation in hypoxia. Although many researchers have demonstrated that GSK3β determines the activation of autophagy during different cytological processes^54–56^, autophagy-regulating GSK3β phosphorylation has been poorly described. Recently, some studies have shown that GSK3β phosphorylation is suppressed by Beclin1 siRNA in renal cells^57^. In combination with our results, we suggest that autophagy directly regulates the phosphorylation of GSK3β and thereby the activation of the Wnt/β-catenin pathway, which is critical for maintaining the myogenesis of C2C12.

Our data demonstrate that epigenetic regulation is critical for the sustained hypoxia-induced inhibitory effect on the myogenesis of myoblasts and that the activation of autophagy is a key step in rescuing the myogenic differentiation of myoblasts, which may provide a prospective strategy for treating myopathies caused by chronic hypoxia. Further studies are needed to explore how autophagy degrades phosphorylated GSK3β.

## Materials and methods

### C2C12 cell line culture and cell hypoxic model

Immortalized murine C2C12 myoblasts obtained from the Cell Bank of the Chinese Academy of Sciences (Shanghai, China), originally derived from C3H mouse leg muscle, were cultured on uncoated 6-well plates at 37 °C and 5% CO_2_ in high-glucose growth medium (4.5 g/l d-glucose) DMEM (GIBCO-BRL, Gaithersburg, MD, USA) supplemented with 10% fetal bovine serum (FBS, GIBCO-BRL), 2 mM l-glutamine (Life Technologies, Rockville, MD, USA), and 1% penicillin–streptomycin (GIBCO-BRL). After reaching confluence, C2C12 myoblasts were induced in differentiation medium (DMEM supplemented with 2% horse serum, 1 mM l-glutamine, 1% penicillin–streptomycin) for 2–3 days.

To establish hypoxia culture conditions, C2C12 myoblasts were placed in a hypoxic (1% O_2_, 5% CO_2_, 37 °C) incubator (Galaxy oxygen control incubator, RS Biotech, Irvine, UK) for 1, 3, 6, 12, 24, 72 h and 7 days. Control cells (Normal group) were incubated for equivalent time frames under normoxic conditions (21% O_2_, 5% CO_2_, 37 °C).

### MTT assay

The viability of C2C12 myoblasts cultured under normoxic or hypoxic conditions was determined using a 3-(4,5-dimethylthiazol-2yl)-2,5-diphenyltetrazolium bromide (MTT) assay carried out for 8 days according to the manufacturer’s protocol (Sigma-Aldrich). Absorbance was determined at 490 nm with a microplate reader (Bio-Tek Instruments, Winooski, VT, USA). Experiments were performed in triplicate.

### 5-Ethynyl-2′-deoxyuridine assays

The proliferation of C2C12 myoblasts cultured under normoxic or hypoxic conditions was determined using an 5-Ethynyl-2′-deoxyuridine (EdU) DNA Proliferation in vitro Detection kit (RiboBio, Guangzhou, China) according to manufacturer´s instructions. Flow cytometry was performed on these cells previously labeled with EdU.

### Apoptosis analysis

The apoptosis of C2C12 myoblasts cultured under normoxic or hypoxic conditions was determined using an apoptosis detection kit (BD Pharmingen) according to the manufacturer’s instructions. Flow cytometry was performed to analyze cell apoptosis, discriminating viable, dead, early apoptotic and late apoptotic cells, by detecting the Annexin-V and propidium iodide (PI) staining and comparing the percentages with the numbers determined for the control group.

### Immunofluorescence staining

C2C12 cells cultured under normoxic or hypoxic conditions until confluence were subsequently maintained in differentiation medium for 3 days to promote myoblast fusion. The cells were then fixed with 4% paraformaldehyde for 15 min at 4 °C, permeabilized with 0.2% Triton X-100 in PBS for 10 min and blocked in 5% normal goat serum for 30 min. For immunofluorescence staining, C2C12 cells were incubated with Myosin (R&D system, 1:100, Minneapolis, MN, USA, MAB4470) or LC3I/II (Cell signal, 1:100, 12741) primary antibodies overnight at 4 °C. Subsequently, they were incubated with Cy3-/FITC-secondary antibody for 1 h at room temperature according to the manufacturer’s instructions.

### qRT-PCR analysis

Total mRNA isolated from C2C12 cells using TRIzol (Invitrogen, Carlsbad, CA, USA) according to the manufacturer’s instructions was reverse-transcribed into cDNA. Real-time PCR detection was carried out using Primescript^TM^ RT master mix (Takara Bio Inc., Otsu, Japan), SYBR Premix Ex TaqTMII (Takara Bio Inc.), and the CFX96 Trademark Real-time PCR detection system (Bio-Rad, California, USA). The expression levels of *MyoG*, *MyoD*, *HDAC1-11*, *LC3*, *Beclin1*, *Atg7*, *Axin2*, and *CCND1* were examined with the primers (Sangon Biotech, China) listed in Supplementary Table 1. *GAPDH* served as a housekeeping gene. Experiments were performed in triplicate.

### Western blotting analysis

C2C12 cells were harvested in RIPA lysis buffer (Beyotime Institute of Biotechnology, Shanghai, China). Whole-cell protein extracts were quantified using the BCA assay, separated by SDS-PAGE 8–12%, and then transferred to PVDF membranes (Millipore, Billerica, MA, USA). Antibodies included Myogenin (Abcam, 1:1000, ab124800, Cambridge, MA, USA), MyoD (Santa Cruz, 1:200, sc-377460), HDAC9 (Abcam, 1:1000, ab59718), HIF1α (Abcam, 1:1000, ab179483), HIF2α (Abcam, 1:1000, ab179825), H3K9 (Abcam, 1:1000, ab32129), H3K14, H3K18, H4K16 (Cell Signaling, 1:1000), LC3I/II (Cell Signal, 1:1000, 12741), Beclin1 (Cell Signal, 1:1000, 3738), Atg5 (Cell Signal, 1:1000, 12994), Atg7 (Cell Signal, 1:1000, 8558), Atg12 (Cell Signal, 1:1000, 4180), p62 (Cell Signaling, 1:1000, 23214), p-GSK3β Ser9 (Cell Signal, 1:1000, 9323), GSK3β (Cell Signal, 1:1000, 12456), and active-β-catenin (Millipore, 1:800, 05–665). Stripped membranes were reprobed with GAPDH (Abcam, 1:4000, ab181602) as a loading control. Signal detection was performed using the ECL Kit (Beyotime Institute of Biotechnology) after incubation with an anti-rabbit or anti-mouse IgG secondary antibody (CoWin Bioscience Co., Beijing, China). Experiments were performed in triplicate.

### Transfection assay

siRNA duplex oligonucleotides against mouse HDAC9 (Gene-Pharma Co, Shanghai, China), β-catenin (Gene-Pharma Co, Shanghai, China), Beclin1 (Gene-Pharma Co), or the negative control (Gene-Pharma Co) were transfected into both normoxic and hypoxic C2C12 myoblasts at a final concentration of 50 nM using siPORTNeoFX. The medium was replaced 8 h later. Experiments were performed in triplicate.

### Small molecule administration

To examine the effect of sodium butyrate (NaB) on the myogenesis of C2C12 cells, C2C12 cells were cultured under hypoxia with or without NaB (200 μM, sigma, 156-54-7). To examine the effect of NaB and Trichostatin A (TSA) on autophagy of C2C12 cells, C2C12 cells were cultured under normoxic conditions with or without NaB (200 μM) and TSA (100 nM, Sigma, 58880-19-6). Rapamycin (100 nM, Sigma, 53123-88-9), NaB (200 μM), 3-MA (5 mM, Sigma, 5142-23-4), and recombinant mouse DKK-1 (100 ng/ml, BioLegend, San Diego, CA, USA, 759604) were added to C2C12 cells cultured under normoxic or hypoxic conditions to examine the relationship between autophagy and the signaling pathways. All the cell samples for qRT-PCR and western blotting were collected according to the manufactures’ instructions.

### Transmission electron microscope (TEM) analysis

C2C12 cells were collected with trypsin, washed with serum-free PBS and primarily fixed in 4% glutaraldehyde and 4% paraformaldehyde (Sigma, pH 7.2) overnight. After washing with PBS, the cells were progressively dehydrated in a graduated series of ethanol solutions (50, 70, 95, and 100%), and then embedded in situ in LX-812 resin (Ladd Research Industries Inc., USA). Subsequently, ultrathin sections (60 nm) were stained with 1% uranyl acetate (30 min) and lead citrate (10 min). The ultrastructure of the cells was then observed using an FEI Tecnai G12 Spirit BioTwin transmission electron microscope (FEI Company, USA) with an accelerating voltage of 100 kV. Digital images were captured on a Veleta CCD camera (Olympus-SIS, Germany).

### Chromatin immunoprecipitation

We used a Chromatin immunoprecipitation (ChIP) assay kit (Merck Millipore, Billerica, MA, USA, 17–371) to confirm the binding between proteins and gene promoters according to the manufacturer’s protocol. Antibodies against HDAC9 (Abcam, ab59718) and polyclonal anti-Histone H3 (acetyl K9) (Abcam, ab10812) were used as detection antibodies, and normal rabbit IgG (Merck Millipore) was used as a negative control. All precipitated DNA samples were analyzed by qRT-PCR, and the results were normalized to the input value. The primers spanning the H3K9-/HDAC9-binding sites at the *Atg7, Beclin1, LC3a*, and *LC3b* promoter (Sangon biotech, China) are listed in Supplementary Table 2.

### Hind limb ischemia model

Male 4-month mice were purchased from the Animal Center of Fourth Military Medical University, Xi’an, China. Sixteen mice were randomly and evenly divided into two groups (eight in each group) to receive either sham or femoral artery ligation surgery. Briefly, femoral artery was ligated at the proximal region just under the inguinal ligament and distally above the profunda femoris branch. A cut was then made between the ligation sites. All of the procedures that involved animals were approved by the Animal use and care committee of the Fourth Military Medical University (license number: SYXK 2012-0023).

### Human subjects

Two arteriosclerosis obliteran patients (male), aged 48 and 53 years respectively were conducted by the Affiliated Hospital of Fourth Military Medical University because of their arteriosclerosis obliterans. Health human muscle samples were collected from two bone fracture patients caused by car accident aged 46 and 50 (male).

The clinical study was approved by the Ethics Committee of the Affiliated Hospital of Fourth Military Medical University, and written informed consent was obtained from all participants prior to sample collection.

### Statistics

The data are presented as the mean ± s.d. Unpaired two-tailed Student’s *t*-tests were applied for comparisons between two groups, and one-way analysis of variance (ANOVA) with a Bonferroni post-test was used for multiple comparisons. All experiments were repeated more than three times, and representative experiments are shown. *P* values <0.05 were considered significant. *P < 0.05, **P < 0.01. Analytic tests were undertaken using SPSS17.0 software.

## Supplementary information

Supplementary Figures

Table S1

Table S2

The muscle tube
