# Supplementary material for: Increase in HDAC9 suppresses myoblast differentiation via epigenetic regulation of autophagy in hypoxia
Source: Cell Death Dis. 2019 Jul 18;10(8):552. doi: 10.1038/s41419-019-1763-2 (PMC6639330; doi:10.1038/s41419-019-1763-2)
Supplement: Supplementary file 1 — Supplementary Figures [file 41419_2019_1763_MOESM1_ESM.pdf]

Increase in HDAC9 suppresses myoblast differentiation via epigenetic regulation of autophagy in hypoxia

Zhang Zhang[2,6], Liqiang Zhang[1,6], You Zhou[4,6], Liya Li[1], Jiangdong Zhao[5], Wen Qin[1], Zuolin Jin[3,*], Wenjia Liu[1,*]

1. State Key Laboratory of Military Stomatology & National Clinical Research Center for Oral Diseases & Shaanxi International Joint Research Center for Oral Diseases, Center for Tissue Engineering, School of Stomatology, Fourth Military Medical University, Xi'an, Shaanxi, 710032, China.
2. General Surgery Department, Tang Du Hospital, Fourth Military Medical University, Xi'an, Shaanxi 710032, China.
3. State Key Laboratory of Military Stomatology & National Clinical Research Center for Oral Diseases & Shaanxi International Joint Research Center for Oral Diseases, Department of Orthodontics, School of Stomatology, Fourth Military Medical University, Xi'an, Shaanxi, 710032, China.
4. State Key Laboratory of Military Stomatology & National Clinical Research Center for Oral Diseases & Shaanxi International Joint Research Center for Oral Diseases, Department of preventive dentistry, Fourth Military Medical University, Xi'an, Shaanxi, 710032, China.
5. Department of Aerospace Biodynamics, Fourth Military Medical University, Xi'an, Shaanxi, 710032, China
6. These authors contributed equally to this work.

*Correspondence: Wenjia Liu, PhD. Phone: +86-29-84776471, Fax: +86-29-83218039.
E-mail: wenjia@xiterm.com; wenjialiu23@163.com.
Zuolin Jin, PhD. Phone: +86-29-84776138
E-mail: zuolinj@163.com

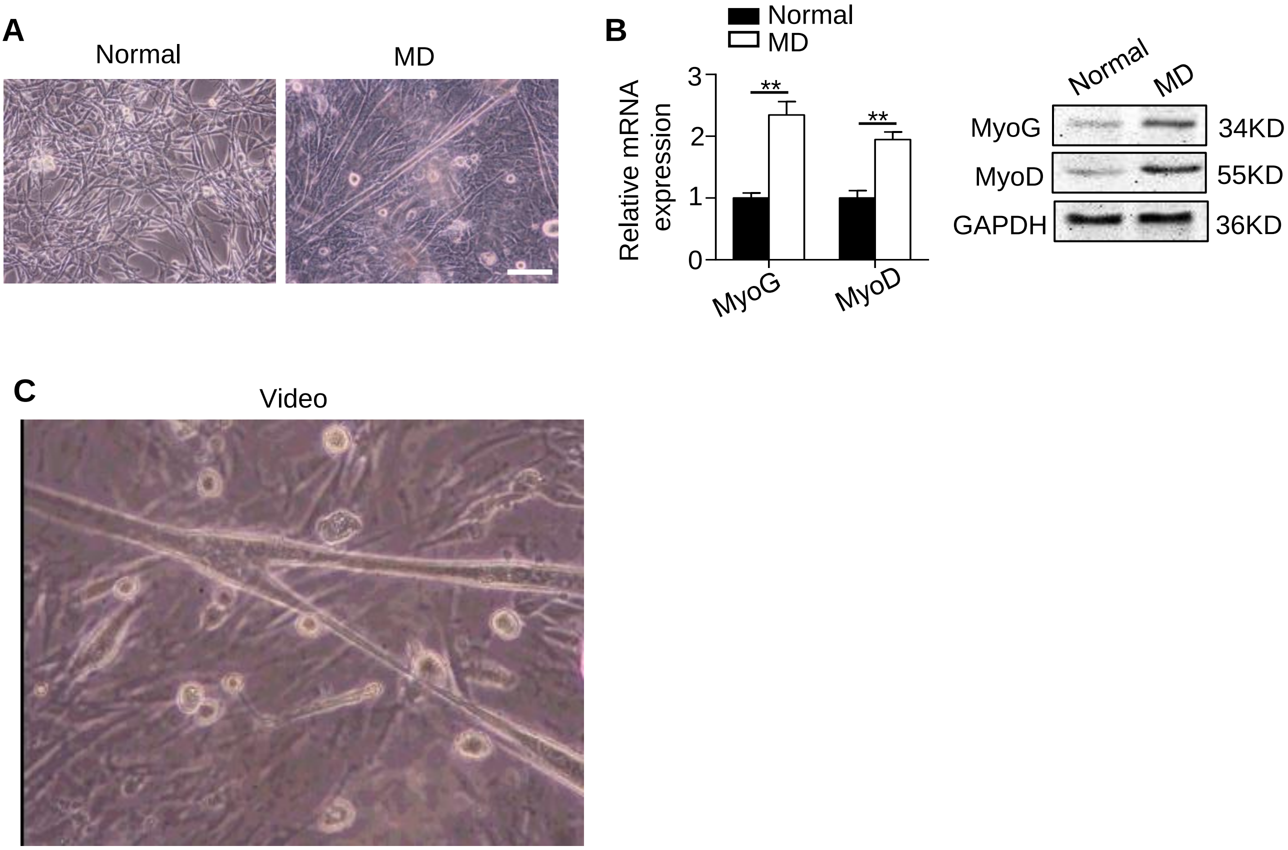

Supplementary Fig S1. Myogenic differentiation of C2C12 cells, related to Fig 1. (**A**) C2C12 cells were induced in normal or myogenic differentiation medium (MD) for 7 days. The cytomorphology of C2C12 cells was observed using an inverted microscope. Scale bar, 50 μm. (**B**) C2C12 cells were induced to undergo myogenic differentiation in MD for 7 days, and the expression levels of MyoG and MyoD in C2C12 cells were examined by qRT-PCR and western blotting. (**C**) A video showing the contraction of the myotube formed by C2C12 cells. Data are the mean ± s.d. of triplicate samples from a representative experiment. *P < 0.05, **P < 0.01. One-way analysis of variance (ANOVA).

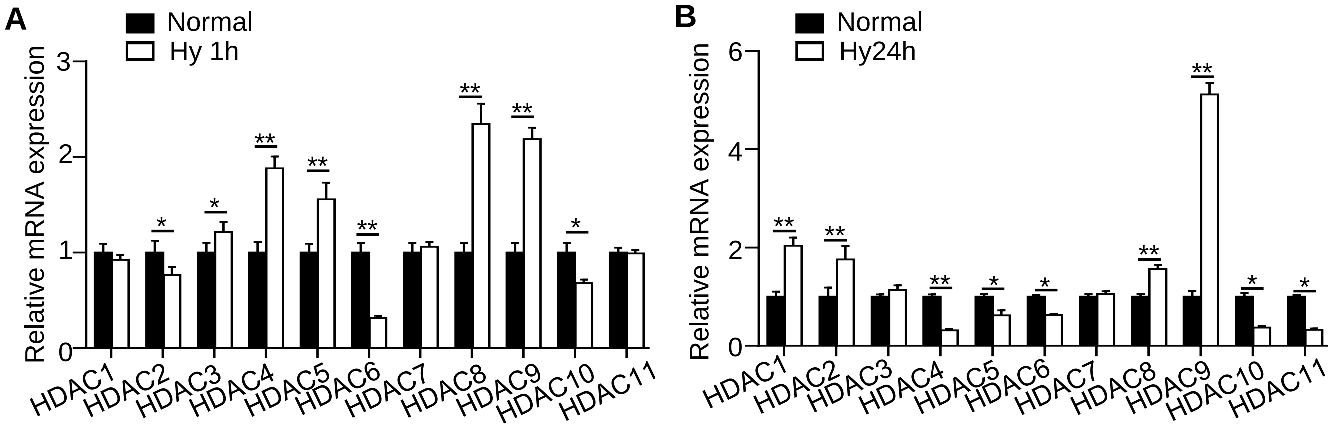

Supplementary Fig S2. Myogenic differentiation of C2C12 cells, related to Fig 2. (A and B) C2C12 cells were cultured under normoxic or hypoxic conditions for 1 hour and 24 hours respectively, and the expression of the histone deacetylase family (*HDAC1-11*) was examined by qRT-PCR. Data are the mean ± s.d. of triplicate samples from a representative experiment. *$P < 0.05$, **$P < 0.01$. One-way analysis of variance (ANOVA).

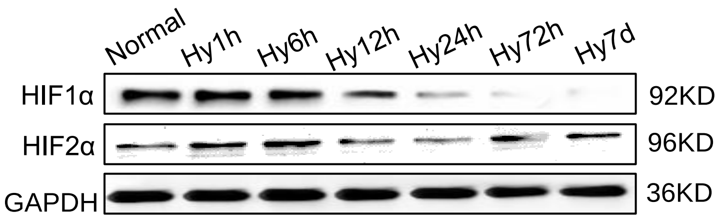

Supplementary Fig S3. The expression of hypoxia-inducible factors in C2C12 cells under hypoxia, related to Fig 2. The expression levels of HIF1α and HIF2α in C2C12 cells at different time points after exposure to hypoxic stimuli were examined by western blotting.

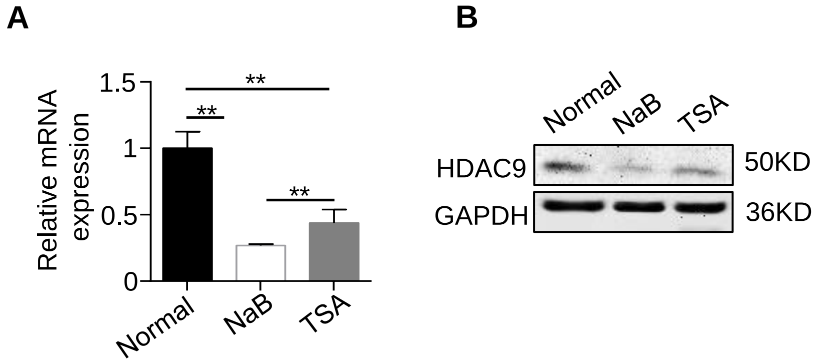

Supplementary Fig S4. The effect of HDAC inhibitors on the regulation of HDAC9 expression, related to Fig 2-6. (**A**, **B**) HDAC9 expression was examined by qRT-PCR and western blotting (**B**). Data are the mean ± s.d. of triplicate samples from a representative experiment. **P* < 0.05, ***P* < 0.01. One-way analysis of variance (ANOVA).

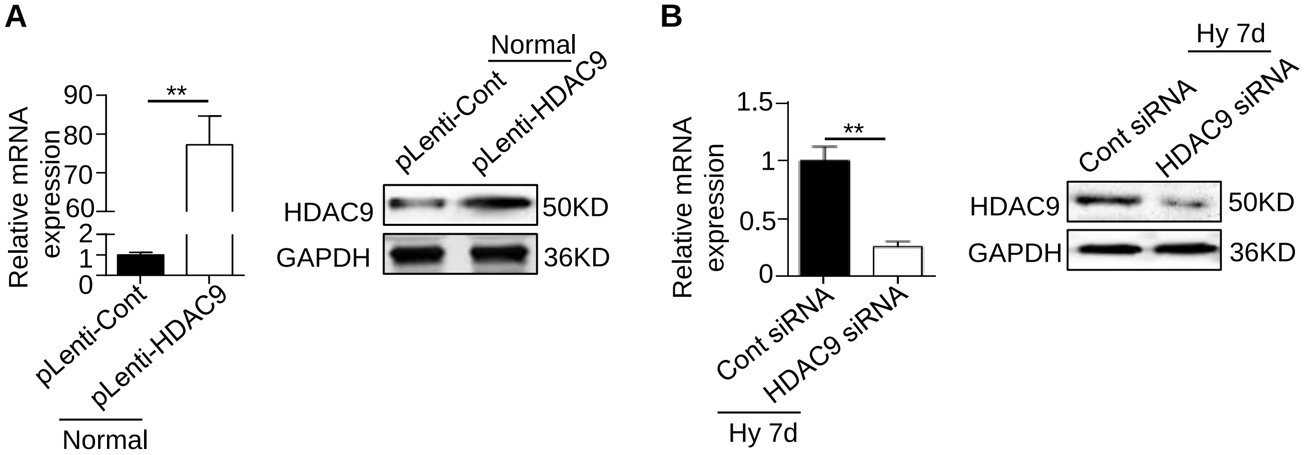

Supplementary Fig S5. The efficiency of lentivirus transduction, related to Fig 3, 4. (**A** and **B**) The efficiency of lentiviral vector on knockdown or overexpressing HDAC9 was confirmed by qRT-PCR and western blotting analysis. The data are presented as means ± s.d. of each independent experiment performed in triplicate. **P* < 0.05, ***P* < 0.01. Unpaired two-tailed Student's t-test.

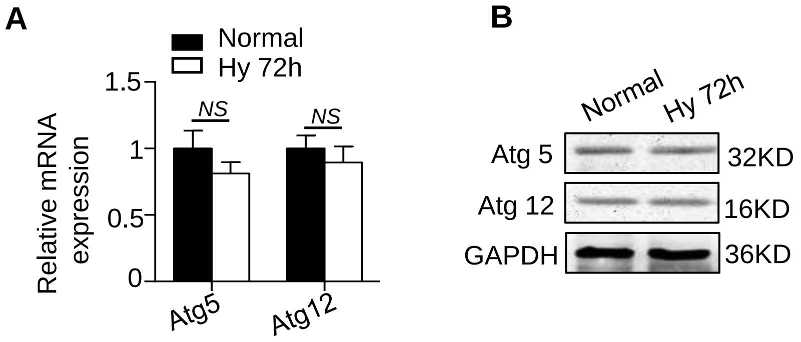

Supplementary Fig S6. The expression of Atg5 and Atg12 in C2C12 cells is not affected by hypoxia, related to Fig 4. (**A, B**) The expression levels of Atg 5 and Atg 12 in C2C12 cells were examined by qRT-PCR (**A**) and western blotting (**B**) after culturing under a normoxic or hypoxic microenvironment for 72 hours. Data are the mean ± s.d. of triplicate samples from a representative experiment. NS, not significant, $P > 0.05$. One-way analysis of variance (ANOVA).

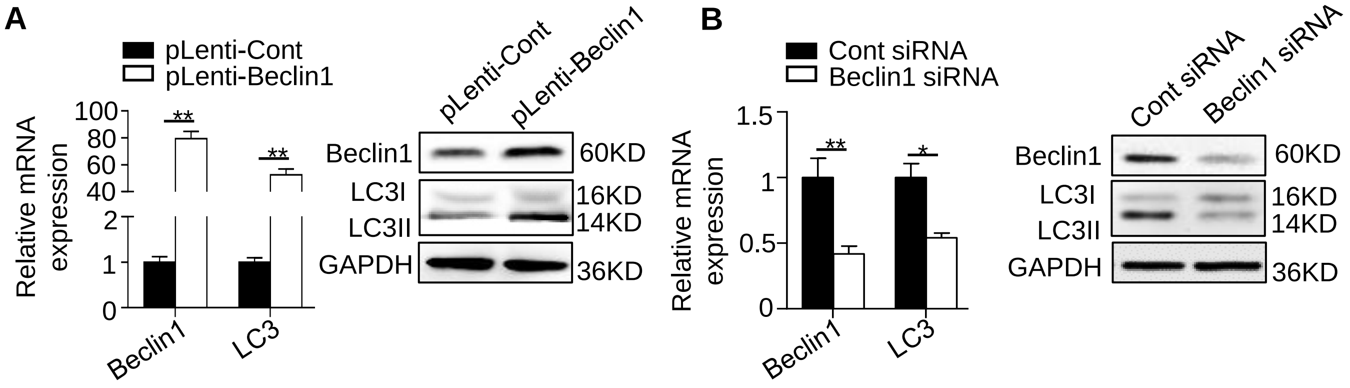

Supplementary Fig S7. The efficiency of lentivirus transduction, related to Fig 5, 6. (**A** and **B**) The efficiency of lentiviral vector on knockdown or overexpressing Beclin1 was confirmed by qRT-PCR and western blotting analysis. The data are presented as means ± s.d. of each independent experiment performed in triplicate. *$P < 0.05$, **$P < 0.01$. Unpaired two-tailed Student's t-test.

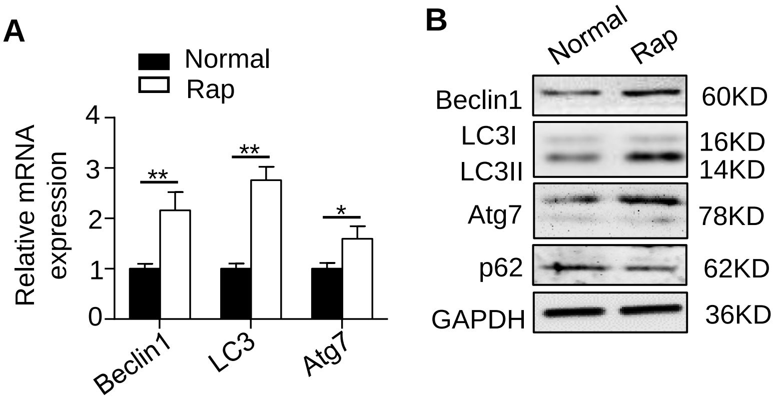

Supplementary Fig S8. Rapamycin activates autophagy in C2C12 cells. (**A** and **B**) C2C12 cells were treated with rapamycin for 72 hours, and autophagy-related gene expression and p62 accumulation in C2C12 cells was examined by qRT-PCR (A) and western blotting (B). Data are the mean ± s.d. of triplicate samples from a representative experiment. *P < 0.05, **P < 0.01. One-way analysis of variance (ANOVA).

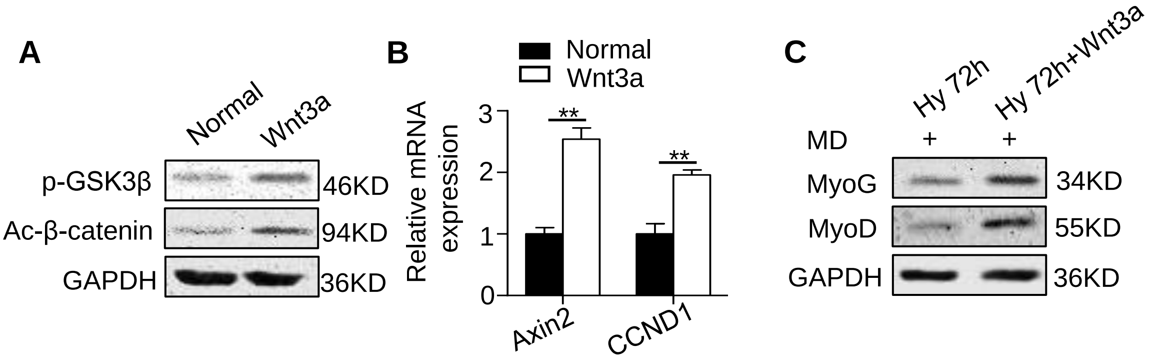

Supplementary Fig S9. Wnt3α activates the canonical Wnt pathway and promotes the myogenic differentiation of C2C12 cells. (**A**) The expression levels of p-GSK3β and ac-β-catenin in C2C12 cells were examined by western blotting after treatment with Wnt3αt for 72 hours. (**B**) The expression levels of downstream genes of the canonical Wnt pathway were examined by qRT-PCR after treatment with Wnt3α for 72 hours. (**C**) The expression levels of MyoG and MyoD were examined by western blotting after culturing in MD medium with or without Wnt3a under hypoxia. Data are the mean ± s.d. of triplicate samples from a representative experiment. **P* < 0.05, ***P* < 0.01. One-way analysis of variance (ANOVA).

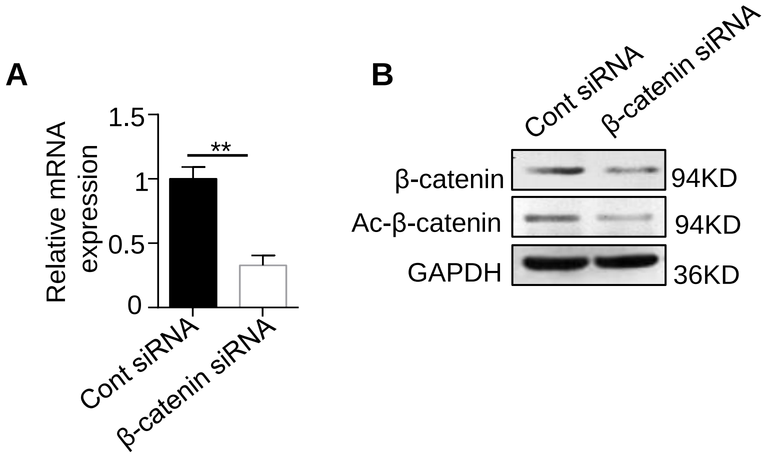

Supplementary Figure S10. The transfection efficiency of β-catenin. (**A**) The expression of β-catenin in C2C2 cells was examined at the mRNA level by qRT-PCR after transfection of β-catenin siRNA for 48 hours. (**B**) Total and activate-β-catenin expression in C2C12 cells were examined at the protein level by western blotting after transfection of β-catenin siRNA for 48 hours. Data are the mean ± s.d. of triplicate samples from a representative experiment. *$P < 0.05$, **$P < 0.01$. Unpaired two-tailed Student’s t-test.
