# Supplementary material for: Increase in HDAC9 suppresses myoblast differentiation via epigenetic regulation of autophagy in hypoxia
Source: Cell Death Dis. 2019 Jul 18;10(8):552. doi: 10.1038/s41419-019-1763-2 (PMC6639330; doi:10.1038/s41419-019-1763-2)
Supplement: Supplementary file 2 — Table S1 [file 41419_2019_1763_MOESM2_ESM.docx]

SupplementaryTable S1

Primer Sequences for Real-Time PCR Analysis

| **Gene name** | **Forward primer** | **Reverse primer** |
| --- | --- | --- |
| *GAPDH* | TGTGTCCGTCGTGGATCTGA | TTGCTGTTGAAGTCGCAGGAG |
| *MyoG* | CTTGCTCAGCTCCCTCAACC | CGATGGACGTAAGGGAGTGC |
| *MyoD* | TACAGTGGCGACTCAGATGC | GTAGTAGGCGGTGTCGTAGC |
| *HADC1* | CCCATGAAGCCTCACCGAAT | CAAACACCGGACAGTCCTCA |
| *HADC2* | TATCCCGCTCTGTGCCCTAC | GAGGCTTCATGGGATGACCC |
| *HADC3* | FCCCCACCAATATGCAGGGTT | CAGAAGCCAGAGGCCTCAAA |
| *HADC4* | GGGAGCAGCATCATGGTTCA | CTAGCAGCGTCAGTGCCTTA |
| *HADC5* | CCGGGAACCATCCTTGGAA | GGGCTACCTCCACCTCCA |
| *HADC6* | CACCGCATTCAGAGGGTTCT | CCTTAAGGTGGGGCCAGAAG |
| *HADC7* | TCCGTGCTGATACCTCTGGC | TCGGGATGCTTGCTGTTGT |
| *HADC8* | CTGCACAAGTTCTCCCCAGG | AGGAAGGGAGGTGTTTCTGGA |
| *HADC9* | GCCTCAGAGCCCAACTTGAA | TTCGGTCACATTCCCAGCAG |
| *HADC10* | GCTTCACAAATCCCGGTTCC | AGCCACAGAATTCTCCCATCA |
| *HADC11* | CTGGCCCATCGTGTACTCAC | GTTGAGATAGCGCCTCGTGT |
| *Beclin1* | CAGTACCAGCGGGAGTATAGTGA | TGTGGAAGGTGGCATTGAAGA |
| *LC3* | CCTGTCCTGGATAAGACCAAGTT | CTCCTGTTCATAGATGTCAGCGAT |
| *Atg5* | CCCTCTTGGGGTACATGTCT | TCGTCCAAACCACACATCTCG |
| *Atg7* | GGTCGTGTCTGTCAAGTGC | CTCCCTGGTGTCCATTAGC |
| *Atg12* | ACAAAGAAATGGGCTGTGGAG | GGAAGGGGCAAAGGACTGAT |
| *Axin2* | CGAGTGTGAGATCCACGGAA | TGGACATGGAATCGTCGGTC |
| *CCND1* | AGGCGGATGAGAACAAGCAG | CCTTGTTTAGCCAGAGGCCG |
| *β-catenin* | TTTCCCAGTCCTTCACGC | GGTCCCAGCAGTACAACG |
