# Supplementary material for: Increase in HDAC9 suppresses myoblast differentiation via epigenetic regulation of autophagy in hypoxia
Source: Cell Death Dis. 2019 Jul 18;10(8):552. doi: 10.1038/s41419-019-1763-2 (PMC6639330; doi:10.1038/s41419-019-1763-2)
Supplement: Supplementary file 3 — Table S2 [file 41419_2019_1763_MOESM3_ESM.docx]

Supplementary Table S2

Primer Sequences for ChIP Analysis

| **Gene name** | **Forward primer** | **Reverse primer** |
| --- | --- | --- |
| *Atg7* | GCAAAGCAAAGGTAAGCAAT | GCTGTTCTGGACCTGTATAC |
| *Beclin1* | AACAAACAAACAACACCCTG | TAGACGGTCCACTCAAGATT |
| *LC3a* | TTTCAGAGGACCTAAGCTTG | AACATAGCATCGGATTTCCT |
| *LC3b* | TCTCCAGACGTCTCCATAAT | TCCTAAATTCTACCCACCCC |
